# Identification of Critical Links Based on Electrical Betweenness and Neighborhood Similarity in Cyber-Physical Power Systems

**DOI:** 10.3390/e26010085

**Published:** 2024-01-19

**Authors:** Jiuling Dong, Zilong Song, Yuanshuo Zheng, Jingtang Luo, Min Zhang, Xiaolong Yang, Hongbing Ma

**Affiliations:** 1 School of Computer and Communication Engineering, University of Science and Technology Beijing, Beijing 100083, China; 2 School of Information Science and Technology, Hainan Normal University, Haikou 571158, China; 3 State Grid Sichuan Economic Research Institute, Chengdu 610041, China; 4 Department of Electronic Engineering, Tsinghua University, Beijing 100084, China

**Keywords:** cyber-physical power system, critical links identification, power flow distribution, electrical betweenness centrality, neighborhood similarity

## Abstract

Identifying critical links is of great importance for ensuring the safety of the cyber-physical power system. Traditional electrical betweenness only considers power flow distribution on the link itself, while ignoring the local influence of neighborhood links and the coupled reaction of information flow on energy flow. An identification method based on electrical betweenness centrality and neighborhood similarity is proposed to consider the internal power flow dynamic influence existing in multi-neighborhood nodes and the topological structure interdependence between power nodes and communication nodes. Firstly, for the power network, the electrical topological overlap is proposed to quantify the vulnerability of the links. This approach comprehensively considers the local contribution of neighborhood nodes, power transmission characteristics, generator capacity, and load. Secondly, in communication networks, effective distance closeness centrality is defined to evaluate the importance of communication links, simultaneously taking into account factors such as the information equipment function and spatial relationships. Next, under the influence of coupled factors, a comprehensive model is constructed based on the dependency relationships between information flow and energy flow to more accurately assess the critical links in the power network. Finally, the simulation results show the effectiveness of the proposed method under dynamic and static attacks.

## 1. Introduction

With the extensive application of information and communication technology (ICT) in the new power system (NPS), the traditional power system has progressively evolved into the cyber-physical power system (CPPS). This transformation represents a deep integration of the communication network (CN) and the power network (PN) [1,2,3]. The intelligent ICT has undoubtedly enhanced the control and operational efficiency of the NPS, but it has also concurrently increased the risk of fault propagation across domains [4,5,6]. Recently, several large-scale blackouts have occurred worldwide, including the blackout in Brazil in 2018, the blackout in Argentina in 2019, and the blackout in Pakistan in 2023 [7,8,9]. While the initial causes of each accident vary, research shows that the fundamental cause of most cascading failures is the tripping of specific transmission lines due to natural or human factors. Therefore, the prompt and precise identification of these vulnerable links is of paramount importance in preventing cascading failures within the NPS. The prompt and accurate identification of these critical links is crucial in preventing the occurrence of cascading failures within the NPS.

Currently, research on identifying critical links in power networks can be divided into two categories based on different analytical perspectives. One category focuses on analyzing a single-sided power network based on physical characteristics. The other category analyzes the contribution of information flow to energy flow from the perspective of a coupled network. Starting from the physical characteristics of the unilateral power network, link vulnerability is measured using various indexes, including electrical distance, electrical centrality, and voltage stability. To dynamically monitor the convergence of power flow and the balance of transfer distribution under link failure conditions, Fang et al. [10] and Shi et al. [11] designed an improved load flow entropy. This method quantitatively analyzes power network components, comprehensively considering the operating limits of the large-scale power system and the characteristics of the components themselves. To identify critical multiple-element branches causing more violations in power systems, Huang et al. [12] and Narimani et al. [13] proposed a method that utilizes the group betweenness centrality and line outage distribution factors, capturing both the topology and the physics of the network. Considering industry-standard security vulnerabilities in the cyber layer, Umunnakwe et al. [14] proposed a cyber-physical betweenness centrality index to evaluate component outages and enhance operators’ resilience. To assess the impact of generator capacity and load level, Wu et al. [15] and Chen et al. [16] proposed the link electrical betweenness based on the equivalent admittance by injecting a unit current source or unit active power between the generator-load node pairs. Although this assessment index overcomes the problem of assuming current flow only along the shortest path in some of the existing models, it ignores the effect of reactive power. Incorporating the real output power as the weight of the load transfer coefficient and taking into account the impact of reactive power, Ding et al. [17] and Bompard et al. [18] introduced a critical link identification index based on electrical betweenness. However, the directionality of the electric current within the links is disregarded. Instead, the absolute values of the currents in different directions are superimposed. In contrast to electrical betweenness, Liu et al. [19] and Bai et al. [20] developed the power flow betweenness model, which takes into account the direction of the electric power flow. Wei et al. [21] and Zang et al. [22] introduced a comprehensive multi-index identification model that employs the game theory weighting method. The model incorporates four key factors: generator output capacity, load size, maximum line transmission capacity, and power transmission characteristics of the new power system. Although this index evaluates link vulnerability from the perspectives of global, local, and operational parameters, it is necessary to assign weights to each sub-metric [23]. To address deficiencies in existing methods, which often overlook the information carried by nodes and insufficiently account for accidental faults in lines, Nan et al. [24] investigated an enhanced maximizing dispersion method based on voltage stability, capacity margin, and real-time fault probability. The above works generally identify critical links in the physical characteristics of a single-sided power network, which can provide a useful reference for the subsequent study of critical links in a PN. Nevertheless, there are still some problems that need to be solved, such as neglecting the contribution of neighboring links within a specific area and the impact of the communication network nodes on the power network nodes in terms of computation time, scheduling, and control. While the methods mentioned above have certain reference significance, they do not simultaneously consider both the global and local operational characteristics of coupled nodes in the power grid.

From the power-communication network (PC) perspective, based on energy flow and information flow, an optimization model of the coupled relationship between power supply and monitoring is developed to analyze the vulnerability of critical links in the PN. In [25], an incidence matrix method was constructed to evaluate the influence of communication network failures (e.g., time delay, bit error, interruption) on the power network. According to [26,27], an optimization formulation was measured in terms of the DC power flow in the presence of interdependence between the communication network and the power network. This formulation quantitatively evaluates the impact of communication component failures on the power network. Although the scholars mentioned above have established coupled network models for vulnerability analysis, the calculation time is relatively long in large power networks. To solve this problem, Nguyen et al. [28] and Xiao et al. [29] developed an intelligent algorithm optimization model for the greedy framework based on the interdependence centrality function to identify critical links. Ti et al. [30] studied a two-layer optimization model of attack and defense games to analyze the impact of cascading failures following circuit breaker and generator failures. Li et al. [31] investigated the controllability evaluation of complex networks via critical nodes and edges, discussing the effects of actual operating conditions on complex network controllability regarding kinetic equations. The above studies show that, compared to unilateral networks, coupled network failure propagation is modeled through a state mapping process, which maps failures in the communication network to failures in the power network and enables a more accurate assessment of link vulnerability in real-life power systems. However, in the above research methods, one should not ignore that links are affected by their own nodes, directly coupled nodes, and the contribution level of neighboring nodes.

Therefore, this paper presents an improved identification algorithm of critical links based on electrical betweenness centrality and neighborhood similarity for the CPPS, which takes into account the power flow transmission structure in the global network, the local influence of two-hop neighborhood information, the operating parameters of the power system, and the direct and indirect interdependencies of coupled networks. To summarize, the main contributions of this paper are as follows:

(1) In a large-scale PN, the vulnerability of links is not only related to the actual occupation of each link by each generation-load node pair but also to the local influence of node neighborhood similarity. Based on the structural and functional characteristics of the PN, the evaluation index of electrical topological overlap is proposed to significantly reduce calculation costs and effectively balance accuracy and efficiency.
(2) In the CN, different types of information devices have varying importance in their functions. By simultaneously considering both the topological characteristics and the functional attributes of information devices, an effective measure of distance closeness centrality is devised, significantly improving recognition accuracy.
(3) In the CPPS, the effects of information flow on energy flow and the impacts of neighboring nodes in the internal network are considered simultaneously. A comprehensive index based on neighborhood electrical betweenness centrality is posed to quantify the vulnerability of links from multiple perspectives.

## 2. Constructing a Coupled Topology Model of the Interdependent Power Communication Network

The CPPS is mainly composed of a CN and a PN, abstracting an unweighted and undirected graph $G(G_{P},G_{C},E_{P-C})$ based on complex network theory [32,33]. In the PN, the power plants, substations, loads, etc. are regarded as power nodes, and the power lines are considered as edges, which can be abstracted as $G_{P}=(V_{p},E_{p})$. $V_{P}=\left\{u_{1},u_{2},\ldots ,u_{n}\right\}$, where $E_{P}=\left\{e_{ij}^{p}\right\}$ are the set of nodes and edges, respectively, in the PN; $u_{i}\in V_{P},(i=1,2\ldots ,n)$ is defined as the *i*th node, and n represents the number of power nodes. In the CN, the wide area measurement system, supervisory control and data acquisition system, synchronized phasor measurement unit, the dispatch center, etc. are regarded as communication nodes, while the communication links are regarded as edges, which can be abstracted as $G_{C}=(V_{C},E_{C})$. $V_{C}=\left\{v_{1},v_{2},\ldots ,v_{m}\right\}$, where $E_{C}=\left\{e_{ij}^{c}\right\}$ are the set of nodes and edges, respectively, in the PN; $v_{i}\in V_{C},(i=1,2\ldots ,m)$ is defined as the *i*th node, and m denotes the number of communication nodes. $E_{PC}=\left\{(u,v)\left|u\in V_{P},v\in V_{C}\right.\right\}$ is used to describe the interdependent coupled edges between the PN and the CN. The coupled network adjacency matrix is shown below:

$$A_{PC}\left(a_{ij}\right)=\left[\begin{array}{ccccccc} a_{11} & a_{12} & \cdots & a_{1n} & a_{1\left(n+1\right)} & \cdots & a_{1\left(n+m\right)}\\ a_{21} & a_{22} & \cdots & a_{2n} & a_{2\left(n+1\right)} & \cdots & a_{2\left(n+m\right)}\\ \vdots & \vdots & \ddots & \vdots & \vdots & \vdots & \vdots \\ a_{n1} & a_{n2} & \cdots & a_{nn} & a_{n\left(n+1\right)} & \cdots & a_{n\left(n+m\right)}\\ a_{\left(n+1\right)1} & a_{\left(n+1\right)2} & \cdots & a_{\left(n+1\right)n} & a_{\left(n+1\right)\times \left(n+1\right)} & \cdots & a_{\left(n+1\right)\times \left(n+m\right)}\\ \vdots & \vdots & \vdots & \vdots & \vdots & \ddots & \vdots \\ a_{\left(n+m\right)1} & a_{\left(n+m\right)2} & \cdots & a_{\left(n+m\right)n} & a_{\left(n+m\right)\times \left(n+1\right)} & \cdots & a_{\left(n+m\right)\times \left(n+m\right)} \end{array}\right]$$

The connection relationship between two nodes in the PC is:

$$a_{ij}=\left\{\begin{array}{l} 1,\;\mathrm{if}\;\mathrm{node}\;i\;\mathrm{is}\;\mathrm{connected}\;\mathrm{to}\;\mathrm{node}\;j\\ 0,\;\mathrm{if}\;\mathrm{node}\;i\;\mathrm{is}\;\mathrm{not}\;\mathrm{connected}\;\mathrm{to}\;\mathrm{node}\;j \end{array}\right.$$

## 3. Building an Identification Model of Critical Links in a Coupled Network

In a coupled network composed of a PN and a CN, the vulnerability of critical links is related to the structure of the unilateral power network and is influenced by dependent communication nodes. Hence, in the coupled network, the critical link identification model proposed in this paper mainly considers three factors. Firstly, in the unilateral power network, it takes into account the local contribution of flow distribution characteristics of the connection itself and the adjacent connections within a certain area. In other words, the higher the flow distribution characteristics of the link, the greater the node degree at both ends, and the lower the degree of neighborhood overlap between neighboring nodes, the higher the vulnerability of the links. Secondly, in a single-sided communication network, the importance of information devices depends not only on their functional characteristics but also on their location in the network topology. Thirdly, in the coupled network, the vulnerability is influenced by the contribution of coupling factors to the link. In other words, the more deeply a link is affected by the communication network under the influence of interdependent coupling, the higher its vulnerability. We establish a comprehensive evaluation index for critical links’ vulnerability based on these three factors.

### 3.1. Constructing a Structure Index Based on Neighborhood Similarity for a Unilateral Power Network

The CPPS utilizes hierarchical scheduling and control technology in its power dispatching system, while the NPS imposes restrictions on power generation, transmission, transformation, and distribution. As a result, the influence of most nodes is limited to local areas. However, in the case of a large-scale NPS, if the local area is too extensive, the computational time complexity will increase. Thus, it is sufficient to consider the contributions of the node and its neighboring nodes within a two-hop range.

**Figure 1:**
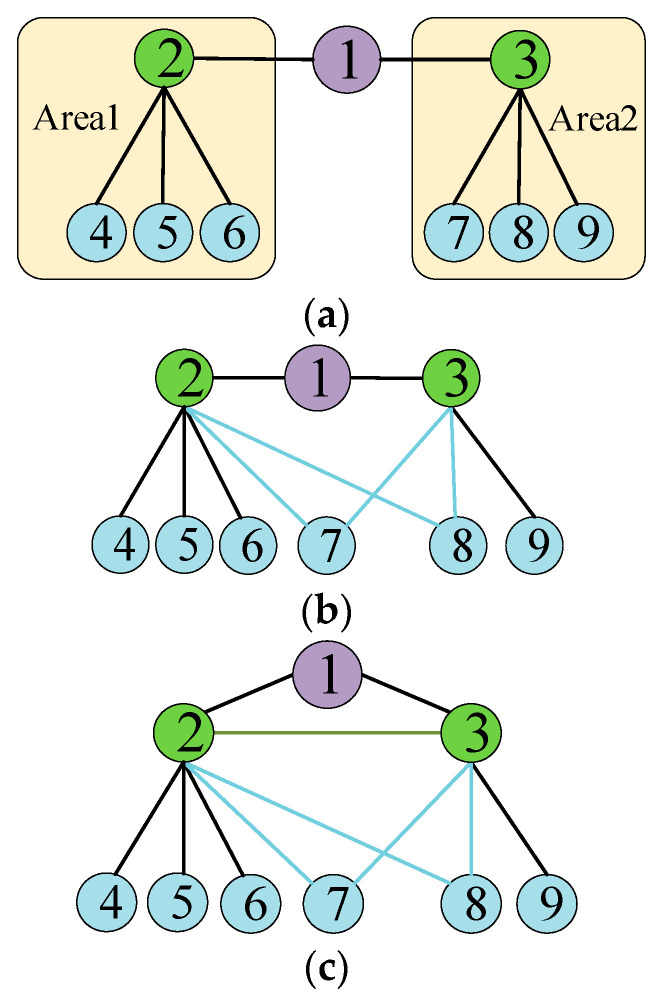
Overlap between the topologies of the neighbors of node 1 in PN. (**a**) There is no overlap between neighborhood nodes of power node 1. (**b**) There is overlap between the neighborhood nodes of power node 1. (**c**) Power node 2 is directly connected to node 3.

As illustrated in Figure 1a, although the degree of node 1 is significantly smaller than that of neighborhood nodes 2 and 3, in terms of network connectivity, node 1 is the only hub for power flow transmission between nodes in area 1 and area 2. Consequently, node 1 has more significance than node 2 and node 3. The hub for flow transmission in Figure 1a is only node 1, while in Figure 1b, there are three nodes (e.g., node 1, node 7, node 8). Consequently, among the three sub-graphs in Figure 1, node 1 in Figure 1a holds the highest importance. Building on this, the identification index of critical links for the NPS is conducted based on neighborhood similarity. Firstly, the Jaccard index based on original similarity in [34] is used to calculate the similarity between any neighboring node *i* and node *j* of node *k* in the PN. The expression is given as:

(1) $$NS\left(i,j\right)=\left\{\begin{array}{ll} \frac{\left|N\left(i\right)\cap N\left(j\right)\right|}{\left|N\left(i\right)\cup N\left(j\right)\right|}, & a_{ij}=0\\ 1, & a_{ij}=1 \end{array}\right.$$

where $a_{ij}=0$ denotes that there is no connecting edge for nodes *i* and *j* in the PN; otherwise, it is a connecting edge.

$N\left(i\right)$ and $N\left(j\right)$ represent the number of neighboring nodes for nodes *i* and *j,* respectively. The range of $NS\left(i,j\right)$ is [0, 1]. $N\left(i\right)\cap N\left(j\right)$ and $N\left(i\right)\cup N\left(j\right)$ represent the intersection and union of the neighboring nodes of node *i* and the neighboring nodes of node *j* in the PN, respectively. It can be seen from Figure 1a, Figure 1b, and Figure 1c, respectively, that $NS\left(2,3\right)$ = 1/7, $NS\left(2,3\right)$ = 3/7, and $NS\left(2,3\right)$ = 1. On this basis, the larger the neighbors of nodes *i* and *j* in the PN, and the lower the degree of network topology overlap between neighbors, the greater the role of PN node *k* in the topology structure. That is, the smaller the value, the more vulnerable the node. When nodes *i* and *j* have a greater number of neighbors in the PN, and the topological overlap among these neighbors is smaller, node *k* exerts a more significant influence on the topological structure of the network, i.e., a smaller value indicates a higher vulnerability for the node. The local vulnerability index of node *k* based on neighborhood similarity (LNS) [34] is defined as follows:

(2) $$LNS_{PN}\left(k\right)=\sum_{i,j\in N_{K}}\left(1-NS\left(i,j\right)\right)$$

where $N_{K}$ is the set of neighborhood nodes for node *k*. Using Equations (1) and (2), we obtain $LNS_{PN}\left(1\right)$ = 6/7 in Figure 1a, $LNS_{PN}\left(1\right)$ = 4/7 in Figure 1b, and $LNS_{PN}\left(1\right)$ = 0 in Figure 1c. According to the above, the larger the value of $LNS_{PN}\left(k\right)$, the stronger the vulnerability of node *k.* The $LNS_{PN}\left(k\right)$ indicator considers the local influence of direct neighboring nodes, while also taking into account the influence of neighboring nodes within two hops. Thus, it can effectively measure the vulnerability of nodes in the NPS.

From a local perspective, the degree of edges in complex networks can be defined by the degree of nodes at both ends [35]. Based on this, the indicator for the topological structures’ local neighborhood similarity (TSLNS) for the link $\left(k_{1},k_{2}\right)$ [24] is formalized as:

(3) $$TSLNS_{PN}\left(k_{1},k_{2}\right)=\frac{LNS\left(k_{1}\right)\times LNS\left(k_{2}\right)}{{\bar{LNS}}^{2}}$$

where $LNS\left(k_{1}\right)$ and $LNS\left(k_{2}\right)$ are the neighborhood similarity of node $k_{1}$ and node $k_{2}$ in the PN, respectively. $\bar{LNS}$ is the average neighborhood similarity of all nodes in the PN.

### 3.2. Constructing Functional Index Based on Electrical Betweenness Centrality for Unilateral Power Network

In the power flow distribution characteristics of the PN, it is a physical fact that the power flow propagates not only along the path with the lowest impedance between buses, but also along all possible paths. To truly reflect the role of each link in the power propagation and influence of different generation-load node pairs, the functional electrical betweenness (FEB) [36], based on the original betweenness in [31,37] for each link, is given as follows:

(4) $$FEB_{PN}\left(k_{1},k_{2}\right)=\left|\sum_{i\in N_{G},j\in N_{L}}w_{ij}P_{k_{1}k_{2}}\left(i,j\right)\right|$$

where $N_{G}$ is the set of generation nodes, and $N_{L}$ is the set of load nodes. $P_{k_{1}k_{2}}\left(i,j\right)$ is the active power generated on the link $\left(k_{1},k_{2}\right)$ when the unit active power $P_{i}=1$ and $P_{j}=-1$ are injected between the generation-load node pairs $\left(i,j\right)$, respectively. The corresponding weight is $W_{ij}=\min\left(S_{i},S_{j}\right)$ in the PN, where $S_{i}$ and $S_{j}$ are the rated generation capacity and the maximum load demand, respectively.

The FEB index can quantify the role of each branch in the power transmission of the entire network. However, it cannot be used to directly compare different networks due to the variation of electrical betweenness values of other nodes with the size of the power network. To address this limitation, the indicator needs to be normalized. The expression for normalization, based on the original betweenness centrality in [37], is calculated as follows:

(5) $$FEBC_{PN}(k_{1},k_{2})=\frac{FEB_{be}(k_{1},k_{2})}{\sum_{i\in N_{G},j\in N_{L}}\sqrt{w_{ij}}}$$

### 3.3. Building a Model Based on Electrical Topological Overlap for Unilateral Power Networks

In a real power network, the vulnerability of links is linked to their own electrical characteristics, distinct from the local influence of the nodes connected to the ends of the power link. Consequently, a comprehensive indicator for electrical topology overlap (ETO) based on electrical betweenness centrality and neighborhood similarity centrality has been proposed. It reflects the occupancy of each link in the power propagation of the entire network, indicating not only the local influence of the link on the topology structure but also its role in the power flow distribution. It is defined as:

(6) $$ETO_{PN}\left(k_{1},k_{2}\right)=\mu FEBC_{PN}(k_{1},k_{2})+\left(1-\mu \right)TSLNS_{PN}\left(k_{1},k_{2}\right)$$

where μ is the weight factor. The larger the value of μ in the power network, the more obvious the distribution characteristics of power flow. The smaller the value of μ, the stronger the local influence of nodes. The value of μ is obtained from the statistical characteristics of two sub-indicators, and its expression is:

(7) $$\mu =\frac{avg(FEBC_{PN})/\mathrm{var}\left(FEBC_{PN}\right)}{avg(FEBC_{PN})/\mathrm{var}\left(FEBC_{PN}\right)+avg(TSLNS_{PN})/\mathrm{var}\left(TSLNS_{PN}\right)}$$

where $avg\left(\cdot \right)$ is the mean function and $\mathrm{var}\left(\cdot \right)$ is the variance function. In the power network, the ETO index takes into account both local structural characteristics and power flow distribution characteristics. Typically, as the ETO value increases, the vulnerability of the link also increases.

### 3.4. Defining a Model Based on Effective Distance Closeness Centrality for Unilateral Communication Networks

In a CPPS, the communication network plays a crucial role in various business functions (e.g., wide area phase measurement, relay protection, and dispatch automation) related to the PN [38,39]. Different information devices hold varying levels of importance in the CN. Therefore, quantifying the vulnerability of nodes in the CN solely based on the topological characteristics of complex networks does not capture the real physical significance. To address this, an effective distance closeness centrality index is proposed, aiming to overcome the one-sidedness of a single indicator by considering both the structural characteristics and functional attributes of information devices. Firstly, the effective length $EL_{st}$ [40] of the edge from node s to node t in the CN is calculated as:

(8) $$EL_{st}=1-\ln\left(\frac{F_{st}}{F_{s}}\right)$$

(9) $$F_{s}=\sum_{h\in H}F_{sh}$$

where $F_{s}$ denotes the sum of all information flows from node s. $F_{st}/F_{s}$ is the proportion of information flows from information node s to node t. $F_{st}$ represents the information flows from node s to node t, which is mainly used to represent the propagation flow of the global mobility network. H is the set of neighboring nodes of node s. Then, the minimum effective path is defined as the path with the minimum sum of effective lengths across traversed edges among all possible paths from node s to node t. $ED_{st}$, the effective distance from node s to node t, is the sum of effective lengths of edges traversed by the minimum effective path. According to the definition of closeness centrality in complex networks, the expression for calculating the effective distance closeness centrality (EDCC) from node s to node t based on the original closeness centrality in [37] is formulated as:

(10) $$\left\{\begin{array}{l} EDCC_{CN}\left(s\right)=\frac{M-1}{\sum_{t=1,t\neq s}^{M}ED_{st}}\\ ED_{st}{=\min}_{\Gamma }\sum_{(u,v)\in \Gamma }EL_{uv} \end{array}\right.$$

where $ED_{st}$ is the effective distance from node s to node t. Γ is the set of all possible paths from node s to node t. $EL_{uv}$ is the effective length from node u to node v in the CN. M represents the total number of communication network nodes. In comparison with the traditional closeness centrality index, EDCC is derived from the incoming traffic of its information node and the total outgoing traffic of neighboring nodes. Therefore, it effectively reflects the local contribution of neighboring nodes. Similar to the definition of power network link index in Section 3.1 of this paper, the expression for the comprehensive index of communication network links is defined as:

(11) $$TSEDCC_{CN}\left(s_{1},s_{2}\right)=\frac{EDCC\left(s_{1}\right)\times EDCC\left(s_{2}\right)}{{\bar{EDCC}}^{2}}$$

### 3.5. Defining a Comprehensive Model Based on Electrical Betweenness Centrality and Neighborhood Similarity

In interconnected power-communication networks, the communication infrastructure plays a crucial role in providing information collection and control functions for the power network. Consequently, the failure of communication nodes, whether due to intrinsic defects or cyber-attacks, can directly or indirectly result in significant disruptions within the power network. For example, if hackers target the control center to trip the circuit breaker, it can result in the disconnection of transmission lines within the power network, leading to load reduction and diminished reliability. Therefore, when identifying the vulnerability of power links within a coupled network, it is possible to separately extract the link vulnerability indicators of the two unilateral networks. Firstly, for the power network, the $ETO_{PN}\left(k_{1},k_{2}\right)$ method in Equation (6) is proposed for identifying internal power links $\left(k_{1},k_{2}\right)$. This methodology is based on the functional electrical betweenness centrality $FEBC_{PN}(k_{1},k_{2})$ in Equation (5) and the topological structures local neighborhood similarity $TSLNS_{PN}\left(k_{1},k_{2}\right)$ in Equation (3). Then, for the communication network, the $TSEDCC_{CN}\left(k_{1},k_{2}\right)$ index in Equation (11) is proposed for identifying internal communication links $\left(k_{1},k_{2}\right)$. Finally, the impact of the coupling factor $\delta_{CN-PN}$ in Equation (13) on the vulnerability of power links is considered. In other words, the communication network monitors whether power links exceed power flow limits and regulates the effects of generator output and load shedding. When quantifying the coupling effects of the CN on the PN, we linearly map the influence values of nodes in the CN to the coupling nodes in the PN. According to the coupling factor, topological structure, and functional characteristics in the CPPS, a new comprehensive vulnerability index of coupled functional features and topological structure (CFTC) is formulated as follows:

(12) $$CFTC_{PC}\left(k_{1},k_{2}\right)=ETO_{PN}\left(k_{1},k_{2}\right)+\delta_{CN-PN}\cdot TSEDCC_{CN}\left(k_{1},k_{2}\right)$$

(13) $$\delta_{CN-PN}=\frac{\sum_{j\in \left[k_{1},k_{2}\right]}\sum_{i}^{m}a_{ij}}{\sum_{j}^{n}\sum_{i}^{m}a_{ij}}$$

where $ETO_{PN}\left(k_{1},k_{2}\right)$ is the electrical characteristic topology redundancy index of the power link $\left(k_{1},k_{2}\right)$. $TSEDCC_{CN}\left(k_{1},k_{2}\right)$ is the effective distance closeness centrality index of the communication link $\left(k_{1},k_{2}\right)$. $\delta_{CN-PN}$ is the impact factor of the CN on the PN. That is, the number of dependent edges for at both ends of the power link $\left(k_{1},k_{2}\right)$ in the coupled network accounts for the proportion of the number of dependent edges for the total power nodes. The larger the value, the stronger the dependence of communication nodes on power nodes.

## 4. Estimating the Performance Evaluation Indexes of Critical Links

To verify the effectiveness and accuracy of the proposed model, this paper establishes the following six fault attack modes based on our methods: CFTC, flow betweenness (FBE), electric betweenness (EBE), and random attack (RA):

(1) Static deliberate attack based on the FBE: critical link values, obtained based on the FBE algorithm, are attacked in sequence in descending order.
(2) Static deliberate attack based on the EBE: critical link values, obtained based on the EBE algorithm, are attacked in sequence in descending order.
(3) Static deliberate attack based on the CFTC comprehensive index: critical link values, obtained based on the CFTC algorithm, are attacked in sequence in descending order.
(4) Dynamic deliberate attack based on the FBE: each time the most critical link is attacked, it is obtained based on the FBE algorithm.
(5) Dynamic deliberate attack based on the EBE: every time the most critical link is attacked, it is obtained based on the EBE algorithm.
(6) Dynamic deliberate attack based on the comprehensive index of the CFTC: every time the most critical link is attacked, it is obtained based on the CFTC algorithm.

**Figure 2:**
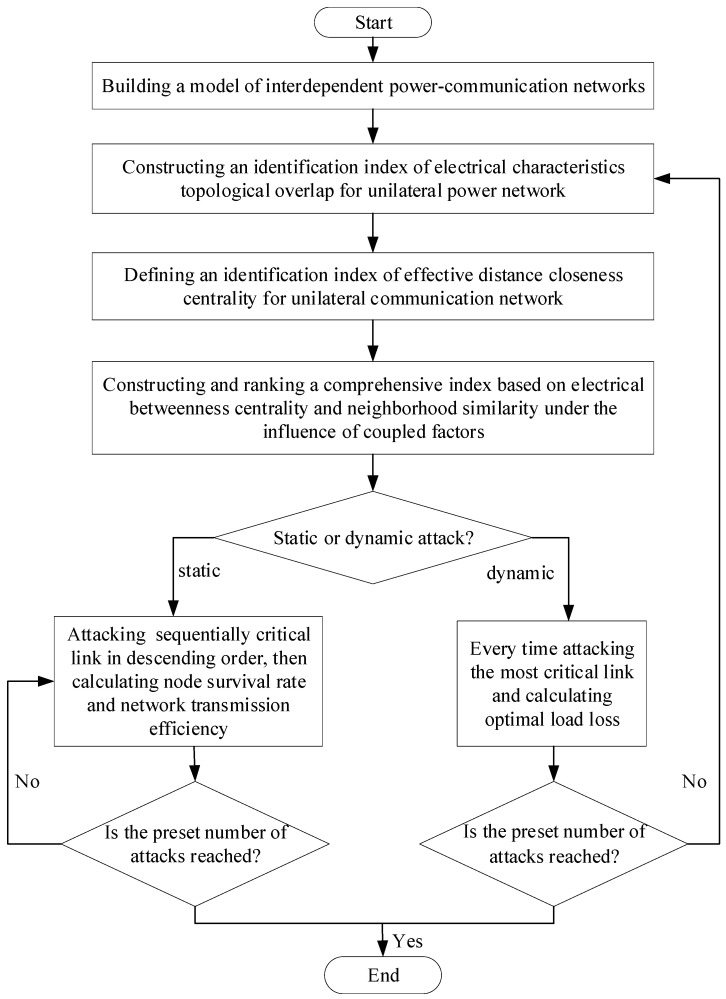
Vulnerability flow chart based on dynamic and static attack modes.

Three evaluation indexes (e.g., node survival rate, network transmission efficiency, optimal load loss) are introduced to quantify the vulnerability of critical links in the PN. Then, a vulnerability analysis is conducted through both static and dynamic attack modes. The specific attack flow is shown in Figure 2.

### 4.1. Node Survival Rate

Generally, the number of remaining nodes in the CPPS is an indication of the stability and robustness of the coupled network after a power link attack. Therefore, the node survival rate is employed to assess the vulnerability of the links in the maximum connectivity subgraph, expressed as follows:

(14) $$R=\frac{{N^{\prime }}_{PN}+{N^{\prime }}_{CN}}{N_{PN}+N_{CN}}$$

where ${N^{\prime }}_{PN}$ and ${N^{\prime }}_{CN}$ represent the remaining number of surviving nodes in the maximum connectivity subgraph for the PN and CN, respectively, when the edges in the PN are attacked, and the cascading fault reaches stability in the coupled network, respectively. $N_{PN}$ and $N_{CN}$ denote the number of nodes in the initial coupled network for the PN and CN, respectively. As the value of R decreases, the proposed method shows a high recognition rate, suggesting that the more severely damaged the network, the fewer nodes remain.

### 4.2. Network Transmission Efficiency

The evaluation of network transmission efficiency involves calculating the average mutual distance between network nodes, which is commonly used to assess node importance in complex networks. However, this criterion mainly focuses on the structural characteristics of the network, overlooking the intrinsic physical properties of the NPS. To align with the actual characteristics of the power system, we modify the shortest distance between two nodes to electrical distance. The expression for network transmission efficiency [41] is as follows:

(15) $$E=\frac{1}{N_{G}N_{L}}\sum_{i\in G}\sum_{j\in L}\frac{\min\left(P_{i},P_{j}\right)}{D_{i,j}}$$

where $N_{G}$ and $N_{L}$ are, respectively, the number of generator nodes and load nodes in the PN. $D_{i,j}$ is the electrical distance of link $\left(i,j\right)$; $\min\left(P_{i},P_{j}\right)$ is the weight factor of the generation-load node pairs, i.e., the maximum transmission power between the node pairs $\left(i,j\right)$ is determined by the smaller value of the actual active power $P_{i}$ and $P_{j}$. In case of a fault, it becomes evident that there is a correlation between increased network transmission efficiency and shorter branch power flow transmission distances between nodes. This leads to a reduction in large-scale power flow transfer, resulting in a lesser impact on the transmission capacity of the PN.

## 5. Case Analysis

**Figure 3:**
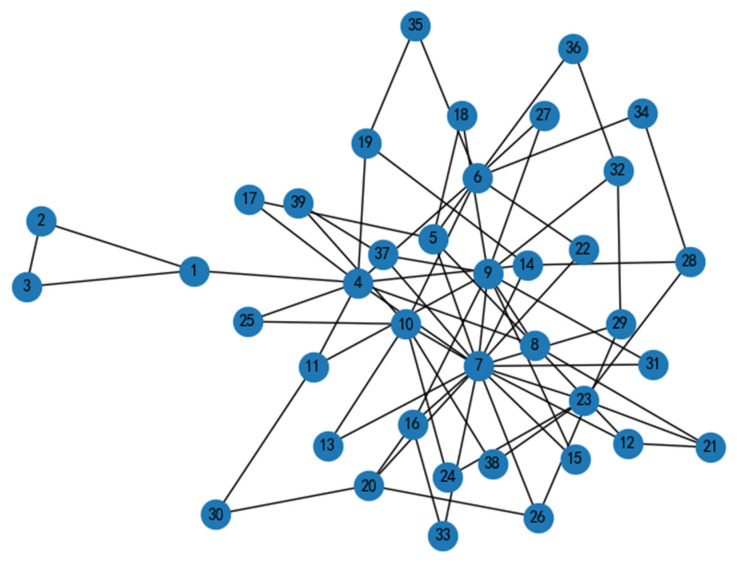
Topology structure of the communication network.

**Figure 4:**
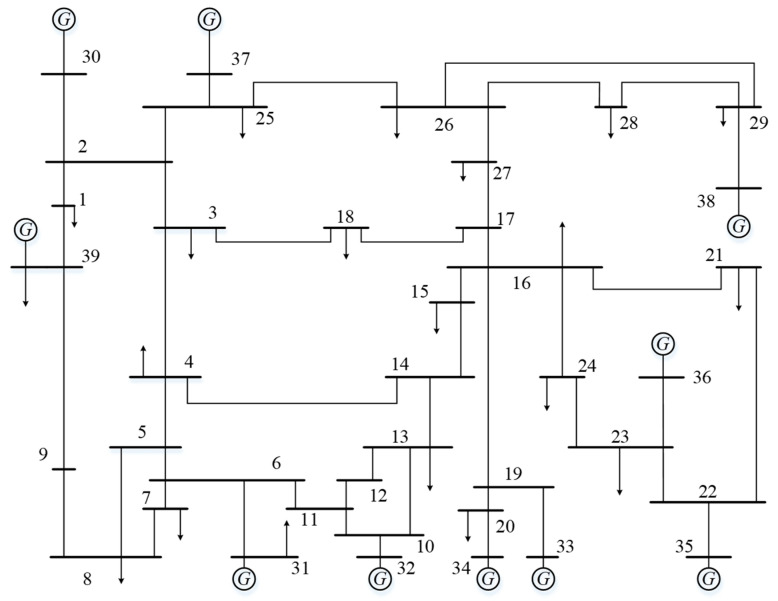
IEEE 39-bus system.

To evaluate the effectiveness of the proposed CFTC algorithm, simulation experiments involving static and dynamic attacks are conducted in interdependent power-communication networks. The power network is modeled using the IEEE 39-bus system, and the communication network adopts a scale-free network structure. The topology of the communication network is illustrated in Figure 3. The IEEE 39-bus system, also known as the 10-machine New England Power System, comprises 39 nodes and 46 power lines. In Figure 4, nodes 31 to 39 are designated as generation nodes. The system operates with a base voltage of 345 KV and a base power of 100 MVA. The communication network is modeled based on the characteristics of scale-free networks. The network is modeled as a scale-free system, characterized by hub nodes with numerous connections, while other nodes have fewer connections. For the communication network model, the scale-free network is interconnected with IEEE 39 nodes from the power network [42]. The communication network model comprises three interconnected nodes, with one designated as the dispatch control center and the other two serving as relay nodes. The optimal load loss of the NPS is calculated during dynamic attacks, and its network transmission efficiency and node survival rate are computed during static attacks.

### 5.1. Analyzing Simulation Results for Critical Links Identification

In accordance with the proposed CFTC algorithm in this paper, the vulnerability index of each power line is initially calculated and ranked in descending order within the NPS. Subsequently, the top 10 links are chosen as the vulnerable links based on the sorting results. The identification of critical links for each of the three testing algorithms (CFTC, FBE, and EBE) is presented in Table 1.

As depicted in Table 1, the CFTC algorithm has identified 4 and 6 as critical links within the top 10 rankings, compared to the FBE and EBE algorithms, respectively. Notably, the essential links identified through these algorithms are the same, but they are ranked differently. For instance, both the traditional FBE and EBE algorithms, along with the CFTC, have recognized links (16-7) and (16-19) with the first and second vulnerability rankings, respectively. From a structural perspective within the NPS, link (16-17) serves as the hub for power flow distribution among the generator units 33 to 36 and other loads and generators. Additionally, the topological overlap of neighboring nodes is minimal. In the event of faults, generator units 33 to 36 become isolated from the main network, leading to the formation of islands and a reduction in network interconnection. The nodes at both ends of link (16-19) have high degrees in the PN and function as the only path for power transmission between generator node 33 and node 34. The abrupt disconnection of links (16-19) leads to the isolation of certain nodes from the main network, causing a power imbalance in the system. The critical link (16-19) identified via the proposed CFTC algorithm aligns with the actual NPS, regardless of whether the analysis involves topology or power flow distribution in the PC.

### 5.2. Verifying the Effectiveness of the CFTC Algorithm

**Figure 5:**
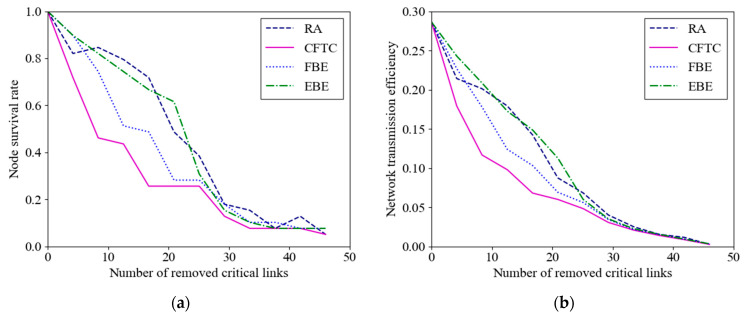
Vulnerability results of different indicators under static edge attack mode: (**a**) vulnerability results of node survival rate; (**b**) vulnerability results of network transmission efficiency.

We conducted attacks on the CPPS in various operating states and illustrated the change curves in node survival rate and network efficiency indexes in Figure 5a,b, respectively. To validate the effectiveness of the CFTC, vulnerability analysis was performed on the CFTC, EBE, and FBE algorithms under static and random attack modes. The analysis was based on node survival rate and network efficiency indexes. To ensure result generalizability, 100 repeated experiments were conducted for each evaluation index, and the results were subsequently averaged. For the deliberate attack experiment, attacks were carried out sequentially, in accordance with the vulnerability ranking results. Figure 5 presents the power network state change curves under different static attacks and random attack modes.

As shown in Figure 5a, with the increase in removed edges, the node survival rate of all four methods in the coupled network continuously decreases under static deliberate attacks and random attacks. However, the rate of decrease for the CFTC is notably faster than that observed with the FBE and EBE algorithms under deliberate attacks and random attacks. For instance, when the removal ratios of critical links are 9%, 27%, 54%, and 81%, the node survival rate of the proposed CFTC algorithm decreases to 71.8%, 43.6%, 25.6%, and 7.7%, respectively. In comparison, the node survival rate obtained using the EBE is reduced to 89.7%, 74.3%, 30.8%, and 7.7%, respectively. The survival rate of the nodes obtained from FBE decreases to 89.7%, 51.3%, 28.2%, and 7.7%, respectively. Both the EBE and FBE algorithms only consider the power flow transmission characteristics of the nodes, ignoring the local influence of neighboring and coupled nodes. Therefore, the CFTC in this paper shows a faster impact on destroying the CPPS during static attacks on the coupled network compared to the EBE and FBE algorithms. The extent of damage to the system resulting from deliberate attacks is typically greater than the impact of random attacks. Building upon this, the effectiveness of the CFTC algorithm is further demonstrated. Figure 5b shows that when critical links are removed from the CPPS at ratios of 9%, 27%, 54%, and 81%, the network efficiency of CFTC decreases to 17.9%, 9.7%, 4.8%, and 0.9%, respectively. In contrast, the network efficiency using the EBE method is reduced to 24.3%, 17.2%, 6%, and 1.5%, respectively. Similarly, the network efficiency of the FBE method decreases to 22.7%, 12.4%, 5.6%, and 0.9%, respectively. Under the same removal ratio, the network efficiency of CFTC decreases more rapidly than that of the EBE and FBE algorithms.

**Figure 6:**
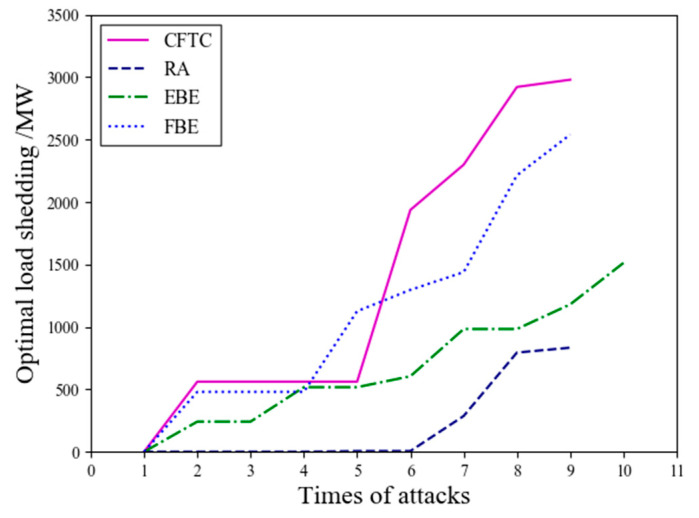
Results of vulnerability for optimal load loss under dynamic attack mode.

In conclusion, the accuracy of vulnerability identification based on the CFTC surpasses that of the other three methods in terms of node survival rate and network efficiency. The static deliberate attack experiments overlook the issue of coupled network structure changes in the link deletion process. Therefore, dynamic deliberate attacks were conducted to further validate the effectiveness of the proposed method. The CFTC method and the other three algorithms were evaluated for optimal load loss separately, followed by a comparative analysis. During the dynamic deliberate attack experiment, the most vulnerable link was selected for each attack, with a hundred attacks per round. Figure 6 depicts the curves of optimal load loss under various dynamic attack modes.

Figure 6 shows that the removal rate of optimal load loss generally increases with the number of attacks. A more detailed analysis is summarized below:

(1) After removing ten critical links, the CFTC algorithm in the CPPS resulted in an optimal load loss of 2979 MW, which is 47.6% lower than the original load data of 6254 MW. In contrast, the EBE and FBE algorithms resulted in load losses of 1512 MW and 2539 MW, with loss ratios of 24.2% and 40.6%, respectively. The analysis shows that the CFTC algorithm has the highest proportion of optimal load loss compared to the other three algorithms.
(2) Specifically, the optimal load loss curve of the EBE method exhibits a slower rise, with an increasing number of attacks, while the optimal load loss curve of the CFTC algorithm demonstrates the fastest increase. In both static and dynamic attack experiments, the CFTC algorithm provides more accurate results for identifying the vulnerability of power links compared to the other three algorithms.
(3) Experimental results indicate that the coupled network in the CPPS demonstrates strong resistance to random attacks but is highly susceptible to deliberate attacks.

## 6. Conclusions

An identification algorithm of critical links based on electrical betweenness centrality and neighborhood similarity has been proposed for the CPPS to promote system robustness against cascading failures. This algorithm takes into account the global influence of dynamic power flow, topological overlap of neighborhood nodes, and coupled factor simultaneously. The following experimental results have been achieved:

(1) In terms of accuracy, when identifying the top 10 ranked set of critical links, the CFTC algorithm exhibits similarity rates of 60% and 40% compared to the EBE and FBE algorithms, respectively.
(2) In static attack scenarios, the CFTC algorithm exhibits lower node survival rates than the FBE and EBE algorithms, with reductions of 17.9% and 17.9% at a 9% power critical link failure rate, and decreases of 7.7% and 30.8% at a 27% failure rate, respectively. Similarly, the CFTC demonstrates decreased network transmission efficiency compared to the FBE and EBE algorithms, with reductions of 4.8% and 6.4% at a 9% power link failure rate, and decreases of 2.6% and 7.5% at a 27% failure rate, respectively.
(3) In dynamic attack scenarios, when disconnecting 10 power critical links, the CFTC algorithm shows load loss reductions of 1467 MW and 440 MW compared to the EBE and FBE algorithms, respectively. Considering the global perspective, deliberate attacks, as a whole, inflict more substantial damage on the coupled network structure compared to random attacks.

The CFTC identifies critical links in the CPPS, but currently lacks a protection strategy for these links. Our upcoming focus will be on adopting a defensive perspective to enhance the vulnerability and robustness of the CPPS by safeguarding vulnerable components.

## Figures and Tables

**Table 1:** Comparison results of critical lines identification through different methods.

Link Ranking	CFTC	FBE	EBE
1	16-17	16-17	16-17
2	16-19	16-19	16-19
3	2-25	17-18	15-16
4	15-16	6-7	14-15
5	2-3	6-11	17-27
6	25-26	16-21	2-25
7	6-11	23-24	26-27
8	3-4	4-14	17-18
9	26-27	8-9	3-18
10	16-21	10-11	2-3

## Data Availability

All data are presented in main text.
